# Meet the Section Editor

**DOI:** 10.2174/1570159X2107230417105921

**Published:** 2023-05-18

**Authors:** Kentaroo Uchida

**Affiliations:** 1 School of Medicine, Kitasato University School of Medicine,Sagamihara, Japan

Dr. Kentaro Uchida is a lecturer at the Kitasato University School of Medicine in Sagamihara, Kanagawa, Japan. He is a member of National Institute of Science and Technology Policy (NISTEP). Fields of expertise include pain, musculoskeletal disorders, neuropeptides, immunology and regenerative medicine. He is the editor and reviewer of several international journals.

## SELECTED PUBLICATIONS

[1] Muneshige, K.; Inahashi, Y.; Itakura, M.; Iwatsuki, M.; Hirose, T.; Inoue, G.; Takaso, M.; Sunazuka, T.; Ohashi, Y.; Ohta, E.; Uchida, K. Jietacin derivative inhibits TNF-α- mediated inflammatory cytokines production *via* suppression of the NF-κB pathway in synovial cells. *Pharmaceuticals (Basel).* **2022**, *16*(1), 5. doi: 10.3390/ph16010005.

[2] Ohashi, Y.; Uchida, K.; Fukushima, K.; Satoh, M.; Koyama, T.; Tsuchiya, M.; Saito, H.; Uchiyama, K.; Takahira, N.; Inoue, G.; Takaso, M. Increased synovial CD14 mRNA expression and proportion of CD14 high subsets in early-stage hip osteoarthritis: propensity matched score analysis. *Int. J. Mol, Sci*., **2022**, *23*(21), 13622. doi: 10.3390/ijms232113622.

[3] Tsukada, A.; Takata, K.; Takano, S.; Ohashi, Y.; Mukai, M.; Aikawa, J.; Iwase, D.; Inoue, G.; Takaso, M.; Uchida, K. Increased NMUR1 expression in mast cells in the synovial membrane of obese osteoarthritis patients. *Int. J. Mol. Sci.,* **2022**, *23*(19), 11237. doi: 10.3390/ijms231911237. PMID: 36232539; PMCID: PMC9570196.

[4] Mukai, M.; Uchida, K.; Hirosawa, N.; Murakami, K.; Inoue, G.; Miyagi, M.; Shiga, Y.; Sekiguchi, H.; Inage, K.; Orita, S.; Suzuki, T.; Matsuura, Y.; Takaso, M.; Ohtori, S. Frozen vein wrapping for chronic nerve constriction injury reduces sciatic nerve allodynia in a rat model. *BMC Neurosci.,* **2022**, *23*(1), 37. doi: 10.1186/s12868-022-00719-7.

[5] Mukai, M.; Uchida, K.; Inoue, G.; Satoh, M,; Miyagi, M.; Yokozeki, Y.; Hirosawa, N,; Matsuura, Y.; Ohtori, S.; Takaso, M. Nerve decompression surgery suppresses TNF-α expression and T cell infiltration in a rat sciatic nerve chronic constriction injury model. *J. Orthop. Res.,* **2022**, *40(*11), 2537-2545. doi: 10.1002/jor.25280.

[6] Ohashi, Y.; Uchida, K,; Fukushima, K.; Satoh, M.; Koyama, T.; Tsuchiya, M.; Saito, H.; Takahira, N.; Inoue, G.; Takaso, M. NGF Expression and Elevation in hip osteoarthritis patients with pain and central sensitization. *Biomed. Res. Int.,* **2021**, *2021,* 9212585. doi: 10.1155/2021/9212585.

[7] Mukai, M.; Uchida, K.; Okubo, T.; Takano, S.; Matsumoto, T.; Satoh, M.; Inoue, G.; Takaso, M. Regulation of tumor necrosis factor-α by peptide Lv in bone marrow macrophages and synovium. *Front. Med. (Lausanne).* **2021**, *8,* 702126. doi: 10.3389/fmed.2021.702126.

[8] Ohashi, Y.; Uchida, K.; Fukushima, K.; Satoh, M.; Koyama, T, Tsuchiya M,; Saito, H.; Uchiyama, K.;Takahira, N.; Inou, G.; Takaso, M. Correlation between CD163 expression and resting pain in patients with hip osteoarthritis: Possible contribution of CD163+ monocytes/macrophages to pain pathogenesis. *J. Orthop. Res.,* **2022**, *40*(6), 1365-1374. doi: 10.1002/jor.25157.

**1570-159X/23** **©2023 Bentham Science Publishers**
